# Free Vibration Characteristics of FG-CNTRC Conical–Cylindrical Combined Shells Resting on Elastic Foundations Using the Haar Wavelet Discretization Method

**DOI:** 10.3390/polym17152035

**Published:** 2025-07-25

**Authors:** Jianyu Fan, Haoran Zhang, Yongqiang Tu, Shaohui Yang, Yan Huang, Zhichang Du, Hakim Boudaoud

**Affiliations:** 1 College of Marine Equipment and Mechanical Engineering, Jimei University, Xiamen 361021, China; xdfjy1990@jmu.edu.cn (J.F.); 202412855070@jmu.edu.cn (H.Z.); shaohuiyang@jmu.edu.cn (S.Y.); heyetodd@jmu.edu.cn (Y.H.); 202061000118@jmu.edu.cn (Z.D.); 2 Fujian Key Laboratory of Energy Cleaning Utilization and Development, Jimei University, Xiamen 361021, China; 3 Key Laboratory of Ocean Renewable Energy Equipment of Fujian Province, Xiamen 361021, China; 4 Équipe de Recherche sur les Processus Innovatifs (EPRI), Université de Lorraine, F-54000 Nancy, France; hakim.boudaoud@univ-lorraine.fr

**Keywords:** carbon nanotube-reinforced composites (CNTRCs), conical–cylindrical combined shells, free vibration, interaction with elastic foundations

## Abstract

Functionally graded carbon nanotube reinforced composites (FG-CNTRCs) are a novel breed of polymer nanocomposite, in which the nonuniform distribution of the carbon nanotube (CNT) reinforcement is adopted to maximize the macro-mechanical performance of the polymer with a lower content of CNTs. Composite conical–cylindrical combined shells (CCCSs) are widely utilized as loading-bearing components in various engineering applications, and a comprehensive understanding of the vibration characteristics of these shells under different external excitations and boundary conditions is crucial for engineering applications. In this study, the free vibration behaviors of FG-CNTRC CCCSs supported by an elastic foundation are examined using the Haar wavelet discretization method (HWDM). First, by means of the HWDM, the equations of motion of each shell segment, the continuity and boundary conditions are converted into a system of algebraic equations. Subsequently, the natural frequencies and modes of the CCCSs are achieved by calculating the resultant algebraic equations. The convergence and accuracy are evaluated, and the results demonstrate that the proposed method has stable convergence, high efficiency, and excellent accuracy. Furthermore, an exhaustive parametric investigation is conducted to reveal the effects of foundation stiffnesses, boundary conditions, material mechanical properties, and geometric parameters on the vibration characteristics of the FG-CNTRC CCCS.

## 1. Introduction

Discovered in 1991, carbon nanotubes (CNTs) have proved to exhibit excellent mechanical, thermal, and electrical properties. As evidenced by prior research, the tensile modulus of CNTs is on the order of 1 TPa, their tensile strength is 100 times greater than steel, and their density is only 1.3 g/cm^3^ [1]. Furthermore, CNTs demonstrate exceptional thermal stability, maintaining structural integrity up to 2800 °C in vacuum conditions. Their thermal conductivity rivals that of diamond [2], while exhibiting electrical current capacity exceeding copper conductors by three orders of magnitude [3]. Consequently, carbon nanotubes (CNTs) are regarded as a promising reinforcement material for advanced polymer composites. Qian [4] et al. experimentally demonstrated that incorporating 1 wt% nanotube fillers into polystyrene matrices yields significant mechanical enhancements, with tensile modulus and strength increasing by 36–42% and 25%, respectively. Similarly, Safadi et al. [5] reported substantial property improvements in CNT-reinforced composites, where the elastic modulus rose from 1.53 Gpa to 3.4 Gpa (122% increase) and strength increased from 19.5 MPa to 30.6 MPa (57% gain) as the CNT volume fraction increased from 0 to 2.5%. The published literature has confirmed that the incorporation of CNTs into the polymer matrix yields great enhancements in the stiffness and strength of the nanocomposite.

Functionally graded CNT-reinforced composites (FG-CNTRCs) are a novel breed of polymer nanocomposite, in which the nonuniform distribution of the CNT reinforcement is adopted to maximize the macro-mechanical performance of the polymer with a lower content of CNTs. Owing to their excellent mechanical properties and broad potential applications in aerospace, ocean, and civil engineering, structures made of FG-CNTRCs—such as beams, plates, and shells—have attracted considerable interest over the last decade. Penna et al. [6] examined the dynamic characteristics of laminated FG-CNTRC beams under hygro-thermal loadings. The free vibration of FG-CNTRC beams subjected to both nonlinear elastic support and uniform temperature rise was studied by Fan and Huang [7]. From the viewpoint of structural safety, Cho and Kim [8] dealt with the numerical procedure to optimally tailor the CNT distribution type in functionally graded CNTRC beams in the thickness direction. Asadi et al. [9] examined the aerothermoelastic characteristics of FG-CNTRC plates in a thermal environment. Mouas et al. [10] studied the vibration behaviors of CNTRC plates with linear and nonlinear distribution patterns of CNTs in a hygro-thermal environment. Cho [11] developed a numerical method to predict the mechanical behaviors of the cracked FG-CNTRC plates. The free vibration of moderately thick FG-CNTRC cylindrical shells under arbitrary edge supports was covered by Mirzaei and Kiani [12]. The vibration behavior of an FG-CNTRC conical shell panel was treated by Cho [13]. An exhaustive review about the mechanical analysis of FG-CNTRC structures was presented by Liew et al. [14].

Conical–cylindrical combined shells (CCCSs), as a typical class of joined shell structures, serve as critical load-bearing components in diverse engineering applications, such as airplane bodies, hydraulic nozzles, submarine hulls, pressure vessels, rockets, and spacecraft. These structures typically operate under highly complex circumstances and withstand various dynamic loads, which may induce undesirable vibrations, noise, or fatigue damage, thereby degrading their system performance. As a consequence, a systematic investigation into the vibrational behavior of these shells under different external excitations and boundary conditions is of great engineering significance to design such structures.

Over the last few decades, considerable contributions have been made to analyze the vibrations of various composite shells. A review about this topic may be found in the literature by Wu et al. [15] and Wang et al. [16]. However, it is noteworthy that the existing studies concentrate on the vibration analysis of fundamental shell configurations (e.g., cylindrical [17,18,19], conical [13,20], and spherical shells [21,22]), with comparatively limited attention given to the joined shells. In comparison to fundamental shell configurations, each shell segment in the joined shell has its own nature description denoted in a different coordinate system, and the interface conditions, such as the continuity conditions and force equilibrium condition, should be matched at the shell–shell junctions. These aspects increase the degree of complexity of the governing equations, resulting in substantial difficulties for accurate vibration analysis. In the last few decades, more and more attention has been paid to the vibration of composite CCCSs, and many analytical and numerical techniques have been presented. Xie et al. [23] analyzed the joined laminated shell structures via the orthogonal polynomial-based spectral collocation method. Employing the spectro-geometric method, Shi et al. [24] treated the thermo-mechanical vibration response of functionally graded conical–cylindrical shell systems. Zang et al. [25] developed a Rayleigh–Ritz-based approach to describe the dynamic evolution of composite conical–cylindrical–conical coupled shells in a thermal environment. Soureshjani et al. [26] employed the generalized differential quadrature method (GDQ) to predict the free vibrational behaviors of sandwich CCCSs subjected to external lateral pressure. Using the same method, Li et al. [27] treated the vibration analysis of porous CCCS under elastically supported boundaries. Xu et al. [28] investigated nonlinear vibration characteristics of fiber-reinforced composite CCCSs under partial bolt looseness using the Rayleigh–Ritz method.

Composite shells frequently interface with soil or other solids in practical implementations. These interaction scenarios commonly occur in marine subsea structures with soil–structure interactions, cylindrical liquid storage vessels in both surface and subsurface installations, and underground pipelines. Among the many factors that may have remarkable and unpredictable effects on the vibration response of such shells are elastic foundations. Consequently, a comprehensive understanding of the vibration characteristics of composite shells coupled with elastic foundations becomes imperative. In recent decades, very few publications have focused on the vibration of composite shells interacting with an elastic foundation. Li et al. [29] studied the vibration properties of a composite sandwich plate on a Pasternak-type elastic foundation. Sofiyev [30] studied the large-amplitude vibration response of functionally graded material (FGM) cylindrical shells on a nonlinear Winkler foundation. The large-amplitude vibration response of nanocomposite cylindrical panels supported by elastic foundations in thermal environments was examined by Shen and Xiang [31] using a two-step perturbation technique. Within the framework of First-order Shear Deformation Theory (FSDT), Tornabene [32] investigated the vibrational characteristics of anisotropic doubly curved shells and panels of revolution with an arbitrary meridian shape interacting with Winkler–Pasternak elastic foundations. Wang et al. [33] employed three-dimensional elasticity theory to analyze the free vibration response of thick open cylindrical shells resting on Pasternak foundations under general boundary conditions. Using a domain decomposition method, Wu et al. [34] dealt with the free vibration of laminated orthotropic conical shells supported by Pasternak foundations. Through a comprehensive literature review, it is evident that most existing studies on the vibration analysis of the composite shells supported by an elastic foundation just focus on the single shell configuration rather than the joined shell.

To the author’s best knowledge, few existing publications have specifically addressed the free vibration behavior of composite CCCSs on an elastic foundation, especially the FG-CNTRC CCCSs. Motivated by this research gap, the primary objective of this study is to establish an accurate and efficient method for analyzing the vibration behavior of the FG-CNTRC CCCSs supported by an elastic foundation.

In this study, we analyze the free vibration responses of FG-CNTRC CCCSs interacting with an elastic foundation employing the Haar wavelet discretization method (HWDM) developed by Majak et al. [35]. The HWDM has gained recognition as an effective analytical tool for various solid mechanics problems [36,37,38] in recent years owing to its merits: implementation simplicity, stable convergence, and remarkable accuracy. The CNTRC CCCS studied here is placed on a Pasternak-type elastic foundation, and the CNTs are considered to be either uniformly dispersed or functionally graded along the thickness direction of the shell structure. The combined shell considered is firstly segmented into conical and cylindrical parts at their junction plane in the meridional direction. Based on the FSDT and Hamilton’s principle, the governing equations of motion are first derived for each individual shell segment resting on the elastic foundation. Subsequently, these shell components are coupled by enforcing the required displacement and force continuous conditions at the junction. Next, the differential equations of motion of each shell segment are transformed into a set of ordinal differential equations (ODEs) via the variable separation technique. The HWDM is subsequently applied to each individual segment, resulting in a system of algebraic equations. In parallel, the method is employed to discretize both the interfacial continuity conditions at the junction and the prescribed boundary conditions at both ends. The natural frequencies and modes of the FG-CNTRC CCCSs supported by the elastic foundation are obtained by solving the resulting algebraic equations. Method validation is achieved through convergence studies and accuracy verification against both the existing literature results and FEM solutions. Furthermore, an exhaustive parametric investigation is conducted to evaluate the influence of the distribution type and volume fraction of CNTs, the stiffness coefficients of the foundation, boundary conditions, and geometric parameters on natural frequencies of the CCCS. Some typical mode shapes of the CCCS are also presented.

The remainder of the present paper is structured as follows. In Section 2, the theoretical model of FG-CNTRC CCCS supported by an elastic foundation is presented. The formulation and implementation of HWDM for the differential equations of motion of the CCCS are detailed in Section 3. In Section 4, the convergence study and accuracy validation of the proposed method are performed. Subsequently, detailed parametric investigations are carried out to examine the effects of material properties, boundary conditions, geometric parameters, and stiffness coefficients of the foundation on the vibrational characteristics of the FG-CNTRC CCCSs. Finally, conclusions are summarized in Section 5.

## 2. Theoretical Model of FG-CNTRC CCCS on Elastic Foundation

### 2.1. System Description

**Figure 1:**
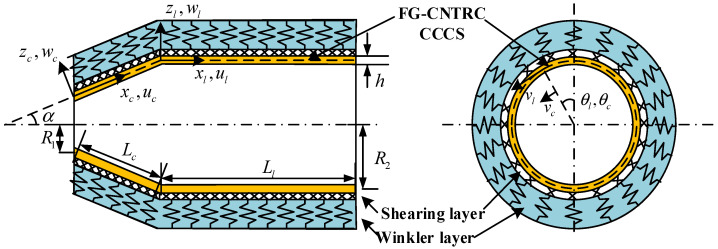
Coordinate system and geometry size of an FG-CNTRC CCCS resting on Pasternak-type foundation.

We consider an FG-CNTRC CCCS supported by an elastic foundation depicted in Figure 1. The ($x_{c}$, $\theta_{c}$, $z_{c}$) coordinate system is adopted to denote the conical shell, in which $x_{c}$ is measured along the cone’s generatrix originating from the vertex, $\theta_{c}$ represents the circumferential angular coordinate, and $z_{c}$ denotes the normal coordinate perpendicular to the shell’s middle surface. The displacement components of the conical shell in the coordinate system are represented by $u_{c}$, $v_{c}$, and $w_{c}$ along the $x_{c}$, $\theta_{c}$, and $z_{c}$ directions, respectively. α denotes the semi-vertex angle of the cone, $R_{1}$ and $R_{2}$, respectively, denote the radii at the small and large ends, and $L_{c}$ specifies the generatrix length of the conical segment. The cylindrical shell is similarly described by the ($x_{l}$, $\theta_{l}$, $z_{l}$) coordinate system with displacement components $u_{l}$, $v_{l}$, and $w_{l}$, respectively. The cylindrical segment has length $L_{l}$ and the combined shell maintains uniform thickness h throughout. The outer surface of the CCCS is continuously supported by a Pasternak elastic foundation model, characterized by the Winkler stiffness modulus $k_{w}$(N/m^3^) and Shear layer stiffness $k_{s}$(N/m). The foundation reaction follows the displacement-dependent relation given by [39]:

(1) $$T_{j}=k_{w}w_{j}-k_{s}\left(\frac{\partial^{2}w_{j}}{\partial x_{j}^{2}}+\frac{\sin\alpha }{R}\frac{\partial w_{j}}{\partial x_{j}}+\frac{1}{R^{2}}\frac{\partial^{2}w_{j}}{\partial \theta_{j}^{2}}\right)$$

where $T_{j}$ denotes the reaction force of the foundation acting on each shell segment. The subscript j is taken to be c or l and denotes a different shell segment, i.e., conical or cylindrical shell. It is noteworthy that when j denotes a cylindrical shell, the semi-vertex angle α is equal to 0. Here, R denotes the radius at an arbitrary point along the shell, where for the cylindrical shell $R=R_{2}$ and for the conical shell $R=R_{1}+x_{c}\sin\alpha$. Notably, the Winkler model represents a particular case of the Pasternak model when $k_{s}=0$.

The considered CCCS is fabricated from a composite material consisting of uniaxially aligned single-walled CNTs embedded in an isotropic polymer matrix. The CNT reinforcement is distributed through the matrix in either functionally graded (FG) patterns or uniform dispersion (UD) along the thickness direction. This study examines four distinct FG distributions (FG-A, FG-V, FG-X, and FG-O) in comparison with the UD configuration. The CNT volume fraction $V_{CNT}$ for each distribution type is mathematically described as follows [40]:

(2) $$V_{CNT}(z)=\left\{\begin{array}{cc} V_{CNT}^{*} & \mathrm{UD}\\ 4V_{CNT}^{*}\left(\frac{\left|z\right|}{h}\right) & \mathrm{FG}\text{-}X\\ 2V_{CNT}^{*}\left(1-2\frac{\left|z\right|}{h}\right) & \mathrm{FG}\text{-}O\\ V_{CNT}^{*}\left(1+2\frac{z}{h}\right) & \mathrm{FG}\text{-}V\\ V_{CNT}^{*}\left(1-2\frac{z}{h}\right) & \mathrm{FG}\text{-}A \end{array}\right.$$

where $V_{CNT}^{*}$ is the total CNT volume fraction expressed as:

(3) $$V_{CNT}^{*}=\frac{w_{CNT}}{w_{CNT}+\left(\rho^{CNT}/\rho^{m}\right)-\left(\rho^{CNT}/\rho^{m}\right)w_{CNT}}$$

where $w_{CNT}$ and $\rho^{CNT}$, respectively, denote the mass fraction and density of CNT, and $\rho^{m}$ represents the mass density of the matrix. It is noteworthy that $V_{CNT}^{*}$ in each distribution type CNTRC CCCS remains consistent.

The effective material properties of FG-CNTRC exhibit continuous variation through the thickness and are determined via the extended rule of mixtures [41]. The detailed description of the evaluation of the effective material properties is expressed as follows:

(4) $$\begin{array}{l} E_{11}\left(z\right)=\eta_{1}V_{CNT}\left(z\right)E_{11}^{CNT}+V_{m}\left(z\right)E^{m},\;\\ \;\frac{\eta_{2}}{E_{22}\left(z\right)}=\frac{V_{CNT}\left(z\right)}{E_{22}^{CNT}}+\frac{V_{m}\left(z\right)}{E^{m}},\;\\ \;\frac{\eta_{3}}{G_{12}\left(z\right)}=\frac{V_{CNT}\left(z\right)}{G_{12}^{CNT}}+\frac{V_{m}\left(z\right)}{G^{m}} \end{array}$$

where $E_{11}^{CNT}$, $E_{22}^{CNT}$, and $G_{12}^{CNT}$, respectively, denote the Young’s and shear modulus of single-walled CNTs, while $E^{m}$ and $G^{m}$ denote the Young’s modulus and shear modulus of the matrix. The efficiency parameters $\eta_{1}$, $\eta_{2}$, and $\eta_{3}$ account for size-dependent material effects and are determined by calibrating the rule of mixtures predictions against molecular dynamics (MD) simulation results [42]. $V_{CNT}\left(z\right)$ and $V_{m}\left(z\right)$, respectively, denote the volume fractions of CNTs and matrix phase, constrained by the relation $V_{CNT}\left(z\right)+V_{m}\left(z\right)=1$. It is evident that the effective material properties exhibit spatial dependence along the thickness direction.

The mass density and Poisson’s ratio of the shell are given by:

(5) $$\rho \left(z\right)=V_{CNT}\left(z\right)\rho^{CNT}+V_{m}\left(z\right)\rho^{m},\;v_{12}\left(z\right)=V_{CNT}\left(z\right)v_{12}^{CNT}+V_{m}\left(z\right)v^{m}$$

where $v^{m}$ and $v_{12}^{CNT}$ denote Poisson’s ratio of the matrix and the CNTs, respectively.

### 2.2. Constitutive Relations and Governing Equations

Preliminarily, the joined shell structure is divided into conical and cylindrical shell components at the junctions. Then, these shell components are connected together to form a whole coupled shell by imposing interface continuity conditions, which require all the displacements to be continuous and the force and moment resultants to be in equilibrium. In the following, the equations of motion of the conical segment subjected to the elastic foundation are first derived based on the FSDT. According to the FSDT, the displacement field is given by [43]:

(6) $$\begin{array}{l} u_{c}\left(x_{c},\theta_{c},z_{c},t\right)=u_{c0}\left(x_{c},\theta_{c},t\right)+z\phi_{cx}\left(x_{c},\theta_{c},t\right)\\ v_{c}\left(x_{c},\theta_{c},z_{c},t\right)=v_{c0}\left(x_{c},\theta_{c},t\right)+z\phi_{c\theta }\left(x_{c},\theta_{c},t\right)\\ w_{c}\left(x_{c},\theta_{c},z_{c},t\right)=w_{c0}\left(x_{c},\theta_{c},t\right) \end{array}$$

where $u_{c0}$, $v_{c0}$ and $w_{c0}$, respectively, denote displacement components of the middle surface along $x_{c}$, $\theta_{c}$ and $z_{c}$ directions. $\phi_{cx}$ and $\phi_{c\theta }$ are the transverse normal rotations; t represents the time variable. The strain–displacement relations are formulated as [43]:

(7) $$\begin{array}{l} \varepsilon_{xx}^{c}=\frac{\partial u_{c0}}{\partial x_{c}},\;\varepsilon_{\theta \theta }^{c}=\frac{\partial v_{c0}}{R\partial \theta_{c}}+\frac{u_{c0}}{R}\sin\alpha +\frac{w_{c0}}{R}\cos\alpha ,\;\gamma_{x\theta }^{c}=\frac{\partial v_{c0}}{\partial x_{c}}+\frac{\partial u_{c0}}{R\partial \theta_{c}}-\frac{v_{c0}}{R}\sin\alpha ,\;\\ \chi_{xx}^{c}=\frac{\partial \phi_{cx}}{\partial x_{c}},\;\chi_{\theta \theta }^{c}=\frac{\partial \phi_{c\theta }}{R\partial \theta_{c}}+\frac{\phi_{cx}\sin\alpha }{R},\;\chi_{x\theta }^{c}=\frac{\partial \phi_{cx}}{R\partial \theta_{c}}+\frac{\partial \phi_{c\theta }}{\partial x_{c}}-\frac{\phi_{c\theta }\sin\alpha }{R},\;\\ \gamma_{xz}^{c}=\frac{\partial w_{c0}}{\partial x_{c}}+\phi_{cx},\;\gamma_{\theta z}^{c}=\frac{\partial w_{c0}}{R\partial \theta_{c}}-\frac{v_{c0}\sin\alpha }{R}+\phi_{c\theta } \end{array}$$

where $\varepsilon_{xx}^{c}$, $\varepsilon_{\theta \theta }^{c}$, and $\gamma_{x\theta }^{c}$ represent the membrane strains in the shell’s middle surface; $\chi_{xx}^{c}$, $\chi_{\theta \theta }^{c}$, and $\;\chi_{x\theta }^{c}$ denote the curvature changes; $\gamma_{xz}^{c}$ and $\gamma_{\theta z}^{c}$ indicate the transverse shear strains.

The constitutive relations between force/moment resultants and strain/curvature components can be denoted in matrix form as [43]:

(8) $$\left[\begin{array}{c} N_{xx}^{c}\\ N_{\theta \theta }^{c}\\ N_{x\theta }^{c}\\ M_{xx}^{c}\\ M_{\theta \theta }^{c}\\ M_{x\theta }^{c} \end{array}\right]=\left[\begin{array}{cccccc} A_{11} & A_{12} & 0 & B_{11} & B_{12} & 0\\ A_{12} & A_{22} & 0 & B_{12} & B_{22} & 0\\ 0 & 0 & A_{66} & 0 & 0 & B_{66}\\ B_{11} & B_{12} & 0 & D_{11} & D_{12} & 0\\ B_{12} & B_{22} & 0 & D_{12} & D_{22} & 0\\ 0 & 0 & B_{66} & 0 & 0 & D_{66} \end{array}\right]\left[\begin{array}{c} \varepsilon_{xx}^{c}\\ \varepsilon_{\theta \theta }^{c}\\ \gamma_{x\theta }^{c}\\ \chi_{xx}^{c}\\ \chi_{\theta \theta }^{c}\\ \chi_{x\theta }^{c} \end{array}\right]\;\left[\begin{array}{c} Q_{\theta z}^{c}\\ Q_{xz}^{c} \end{array}\right]=\kappa_{s}\left[\begin{array}{cc} A_{44} & 0\\ 0 & A_{55} \end{array}\right]\left[\begin{array}{c} \gamma_{\theta z}^{c}\\ \gamma_{xz}^{c} \end{array}\right]$$

where $\kappa_{s}$ represents the shear correction factor, taken as 5/6. $N_{xx}^{c}$, $N_{\theta \theta }^{c}$, and $N_{x\theta }^{c}$ denote the in-place axial, circumferential, and shearing force resultants per unit length, respectively, while $M_{xx}^{c}$, $M_{\theta \theta }^{c}$, and $M_{x\theta }^{c}$ correspond to the bending and twisting moment resultants. $Q_{\theta z}^{c}$ and $Q_{xz}^{c}$ indicate the transverse shear force resultants. The superscript c denote the corresponding variables for the conical shell segment. The extensional stiffness $A_{ij}$, the bending stiffness $D_{ij}$ and the extensional-bending coupling stiffness $B_{ij}$ are obtained through the following integrals:

(9) $$\left(A_{ij},\;B_{ij},\;D_{ij}\right)=\int_{-h/2}^{h/2}\left(Q_{ij}(z),\;zQ_{ij}(z),\;z^{2}Q_{ij}(z)\right)dz$$

in which the elastic constants $Q_{ij}(z)$ are dependent on the normal coordinate z and are given by:

(10) $$Q_{11}=\frac{E_{11}}{1-v_{12}v_{21}},\;Q_{12}=\frac{\nu_{21}E_{11}}{1-v_{12}v_{21}},\;Q_{22}=\frac{E_{22}}{1-v_{12}v_{21}},\;Q_{55}=Q_{66}=G_{12},\;Q_{44}=1.2G_{12}$$

Here, $E_{11}$, $E_{22}$, $G_{12}$, and $v_{12}$ of FG-CNTRC are expressed in Equations (4) and (5). The Poisson’s ratio $v_{21}$ is obtained by the relation $v_{12}E_{22}=v_{21}E_{11}$.

Through implementation of Hamilton’s principle, the governing equations of motion for the FG-CNTRC conical shell on an elastic foundation are derived within the FSDT framework, expressed in terms of force and moment resultants as follows [43]:

(11) $$\begin{array}{l} \frac{\partial N_{xx}^{c}}{\partial x}+\frac{1}{R}\frac{\partial N_{x\theta }^{c}}{\partial \theta }+\frac{\sin\alpha }{R}\left(N_{xx}^{c}-N_{\theta \theta }^{c}\right)=I_{0}\frac{\partial^{2}u_{c0}}{\partial t^{2}}+I_{1}\frac{\partial^{2}\phi_{cx}}{\partial t^{2}}\\ \frac{\partial N_{x\theta }^{c}}{\partial x}+\frac{1}{R}\frac{\partial N_{\theta \theta }^{c}}{\partial \theta }+\frac{2\sin\alpha }{R}N_{x\theta }^{c}+\frac{\cos\alpha }{R}Q_{\theta z}^{c}=I_{0}\frac{\partial^{2}v_{c0}}{\partial t^{2}}+I_{1}\frac{\partial^{2}\phi_{c\theta }}{\partial t^{2}}\\ \frac{\partial Q_{xz}^{c}}{\partial x}+\frac{1}{R}\frac{\partial Q_{\theta z}^{c}}{\partial \theta }+\frac{\sin\alpha }{R}Q_{xz}^{c}-\frac{\cos\alpha }{R}N_{\theta \theta }^{c}-k_{w}w_{c0}+k_{s}(\frac{\partial^{2}w_{c0}}{\partial x_{c}^{2}}+\frac{\sin\alpha }{R}\frac{\partial w_{c0}}{\partial x_{c}}\\ +\frac{1}{R^{2}}\frac{\partial^{2}w_{c0}}{\partial \theta_{c}^{2}})=I_{0}\frac{\partial^{2}w_{c0}}{\partial t^{2}}\\ \frac{\partial M_{xx}^{c}}{\partial x}+\frac{1}{R}\frac{\partial M_{x\theta }^{c}}{\partial \theta }+\frac{\sin\alpha }{R}\left(M_{xx}^{c}-M_{\theta \theta }^{c}\right)-Q_{xz}^{c}=I_{1}\frac{\partial^{2}u_{c0}}{\partial t^{2}}+I_{2}\frac{\partial^{2}\phi_{cx}}{\partial t^{2}}\\ \frac{\partial M_{x\theta }^{c}}{\partial x}+\frac{1}{R}\frac{\partial M_{\theta \theta }^{c}}{\partial \theta }+\frac{2\sin\alpha }{R}M_{x\theta }^{c}-Q_{\theta z}^{c}=I_{1}\frac{\partial^{2}v_{c0}}{\partial t^{2}}+I_{2}\frac{\partial^{2}\phi_{c\theta }}{\partial t^{2}} \end{array}$$

where $\left\{I_{0},I_{1},I_{2}\right\}=\int_{-h/2}^{h/2}\rho (z)\left\{1,z,z^{2}\right\}dz$ represent the inertia terms. It can be noted that the reaction of the foundation acting on the shell is introduced into the third equation of Equation (11).

In the following, we will use the variable separation technique to derive the simplified governing equation of motion. Owing to the axisymmetric characteristics of the shell geometry, the displacement and rotation components can be expressed through the following harmonic expansion [44]:

(12) $$\begin{array}{l} u_{c0}\left(\varphi ,\theta ,t\right)=U^{c}\left(x\right)\cos\left(n\theta \right)e^{i\omega t},\;v_{c0}\left(\varphi ,\theta ,t\right)=V^{c}\left(x\right)\sin\left(n\theta \right)e^{i\omega t},\\ w_{c0}\left(\varphi ,\theta ,t\right)=W^{c}\left(x\right)\cos\left(n\theta \right)e^{i\omega t},\;\phi_{cx}\left(\varphi ,\theta ,t\right)=\Phi_{x}^{c}\left(x\right)\cos\left(n\theta \right)e^{i\omega t},\\ \phi_{c\theta }\left(\varphi ,\theta ,t\right)=\Phi_{\theta }^{c}\left(x\right)\sin\left(n\theta \right)e^{i\omega t} \end{array}$$

where ω represents the natural angular frequency of vibration, while n denotes the circumferential wave number and is a non-negative integer. $U^{c}\left(x\right)$, $V^{c}\left(x\right)$, $W^{c}\left(x\right)$, $\Phi_{x}^{c}\left(x\right)$ and $\Phi_{\theta }^{c}\left(x\right)$ describe the spatial variation of displacement and rotation components to be determined and $e^{i\omega t}$ indicates the time-dependent component. By substituting Equations (6)–(10) and (12) into Equation (11) and eliminating trigonometric terms, the governing equations reduce to:

(13) $$\begin{array}{l} G_{111}U^{c}+G_{112}\frac{dU^{c}}{dx}+G_{113}\frac{d^{2}U^{c}}{dx^{2}}+G_{121}V^{c}+G_{122}\frac{dV^{c}}{dx}+G_{131}W^{c}+G_{132}\frac{dW^{c}}{dx}+G_{141}\Phi_{x}^{c}\\ +G_{142}\frac{d\Phi_{x}^{c}}{dx}+G_{143}\frac{d^{2}\Phi_{x}^{c}}{dx^{2}}+G_{151}\Phi_{\theta }^{c}+G_{152}\frac{d\Phi_{\theta }^{c}}{dx}+I_{0}\omega^{2}R^{2}U^{c}+I_{1}\omega^{2}R^{2}\Phi_{x}^{c}=0\\ G_{211}U^{c}+G_{212}\frac{dU^{c}}{dx}+G_{221}V^{c}+G_{222}\frac{dV^{c}}{dx}+G_{223}\frac{d^{2}V^{c}}{dx^{2}}+G_{231}W^{c}+G_{241}\Phi_{x}^{c}+G_{242}\frac{d\Phi_{x}^{c}}{dx}\\ +G_{251}\Phi_{\theta }^{c}+G_{252}\frac{d\Phi_{\theta }^{c}}{dx}+G_{253}\frac{d^{2}\Phi_{\theta }^{c}}{dx^{2}}+I_{0}\omega^{2}R^{2}V^{c}+I_{1}\omega^{2}R^{2}\Phi_{\theta }^{c}=0\\ G_{311}U^{c}+G_{312}\frac{dU^{c}}{dx}+G_{321}V^{c}+G_{331}W^{c}+G_{332}\frac{dW^{c}}{dx}+G_{333}\frac{d^{2}W^{c}}{dx^{2}}+G_{341}\Phi_{x}^{c}+G_{342}\frac{d\Phi_{x}^{c}}{dx}\\ +G_{351}\Phi_{\theta }^{c}+I_{0}\omega^{2}R^{2}W^{c}=0\\ G_{411}U^{c}+G_{412}\frac{dU^{c}}{dx}+G_{413}\frac{d^{2}U^{c}}{dx^{2}}+G_{421}V^{c}+G_{422}\frac{dV^{c}}{dx}+G_{431}W^{c}+G_{432}\frac{dW^{c}}{dx}+G_{441}\Phi_{x}^{c}\\ +G_{442}\frac{d\Phi_{x}^{c}}{dx}+G_{443}\frac{d^{2}\Phi_{x}^{c}}{dx^{2}}+G_{451}\Phi_{\theta }^{c}+G_{452}\frac{d\Phi_{\theta }^{c}}{dx}+I_{1}\omega^{2}R^{2}U^{c}+I_{2}\omega^{2}R^{2}\Phi_{x}^{c}=0\\ G_{511}U^{c}+G_{512}\frac{dU^{c}}{dx}+G_{521}V^{c}+G_{522}\frac{dV^{c}}{dx}+G_{523}\frac{d^{2}V^{c}}{dx^{2}}+G_{531}W^{c}+G_{541}\Phi_{x}^{c}+G_{542}\frac{d\Phi_{x}^{c}}{dx}\\ +G_{551}\Phi_{\theta }^{c}+G_{552}\frac{d\Phi_{\theta }^{c}}{dx}+G_{553}\frac{d^{2}\Phi_{\theta }^{c}}{dx^{2}}+I_{1}\omega^{2}R^{2}U^{c}+I_{2}\omega^{2}R^{2}\Phi_{x}^{c}=0 \end{array}$$

where $G_{ijk}$(i,j=1~5,k=1~3) denote the constants given in Appendix A.

Through this formulation, the partial differential equations governing the motion of the conical segment are transformed into a system of ODEs, which will subsequently be discretized via the HWDM presented in the following section. It is noteworthy that the cylindrical shell configuration emerges as a limiting case of the conical shell when the semi-vertex angle approaches zero. Therefore, the governing equations in Equation (13) can also be used for the cylindrical shell segment.

### 2.3. Continuity and Boundary Conditions

To couple the two shell segments together, the displacement and force continuous conditions must be satisfied at the junction. Accounting for curvature variation, the displacement continuity and force and moment equilibrium at the junction interface of two segments for the CCCS are expressed as [45]:

(14) $$\begin{array}{l} U^{l}(0)=U^{c}(L_{c})\cos\alpha -W^{c}(L_{c})\sin\alpha ,\;V^{l}(0)=V^{c}(L_{c}),\\ W^{l}(0)=U^{c}(L_{c})\sin\alpha +W^{c}(L_{c})\cos\alpha ,\;\Phi_{x}^{l}(0)=\Phi_{x}^{c}(L_{c}),\;\Phi_{\theta }^{l}(0)=\Phi_{\theta }^{c}(L_{c})\;\\ N_{xx}^{l}(0)=N_{xx}^{c}(L_{c})\cos\alpha -Q_{xz}^{c}(L_{c})\sin\alpha ,\;N_{x\theta }^{l}(0)=N_{x\theta }^{c}(L_{c}),\;\\ Q_{xz}^{l}(0)=N_{xx}^{c}(L_{c})\sin\alpha +Q_{xz}^{c}(L_{c})\cos\alpha ,\;M_{xx}^{l}(0)=M_{xx}^{c}(L_{c}),\;M_{x\theta }^{l}(0)=M_{x\theta }^{c}(L_{c}) \end{array}$$

where the displacement and force variables with superscript l denote the corresponding variables for the cylindrical shell segment.

In the present analysis, three distinct boundary conditions are implemented at each end of the combined shell structure, namely the fully clamped (C), the simple-diaphragm (SD), and the free (F). The mathematical expressions for these boundary conditions are formulated as follows:

Fully clamped boundary condition (C):

(15) $$U=V=W=\Phi_{x}=\Phi_{\theta }=0\;\mathrm{at}\;x_{c}=0\;\mathrm{or}\;x_{l}=L_{l}$$

Simple-diaphragm boundary condition (SD):

(16) $$N_{xx}=V=W=M_{xx}=M_{x\theta }=0\;\mathrm{at}\;x_{c}=0\;\mathrm{or}\;x_{l}=L_{l}$$

Free boundary condition (F):

(17) $$N_{xx}=N_{x\theta }=Q_{x}=M_{xx}=M_{x\theta }=0\;\mathrm{at}\;x_{c}=0\;\mathrm{or}\;x_{l}=L_{l}$$

## 3. Solution Methodology

The HWDM has been established as a robust numerical technique for solving various classes of differential and integro-differential equations [46]. In this section, we develop the HWDM framework specifically for analyzing the governing equations of motion of FG-CNTRC CCCSs supported by elastic foundations.

### 3.1. Haar Wavelet and Its Integrals

The Haar wavelet represents the simplest orthonormal wavelet system possessing compact support. Mathematically, the Haar wavelet family $h_{i}(\xi )$ is defined as a piecewise constant function on the interval ξ∈[0,1], taking values ±1 on specific subintervals and zero elsewhere, with the general form expressed as [47,48]:

(18) $$h_{i}\left(\xi \right)=\left\{\begin{array}{c} 1\;\xi \in \left[\xi_{1}(i),\xi_{2}(i)\right)\;\\ -1\;\xi \in \left[\xi_{2}(i),\xi_{3}(i)\right)\;\\ \;0\;elsewhere \end{array}\right.$$

where $\xi_{1}(i)=k/m$, $\xi_{2}(i)=\left(k+0.5\right)/m$ and $\xi_{3}(i)=\left(k+1\right)/m$. $m=2^{j}$ $\left(j=0,1,\ldots ,J\right)$ denotes the factor of scale, where J represents the maximal level of resolution. k denotes the translation parameter (k=0,1,…,m−1). The index i is defined as i=m+k+1, while the maximal value is taken as i=2M and the minimal value is taken as i=2 when k=0 and m=1. Here, $M=2^{J}$. It should be noted that the case i=1 corresponds to the scaling function $h_{1}(\xi )\equiv 1$.

Consider a square-integrable finite function f(ξ) in the interval [0, 1]. While f(ξ) admits an infinite-term Haar wavelet series expansion, the practical implementation requires truncation to finite terms. Thus, f(ξ) can be approximated by the truncated series as:

(19) $$f(\xi )=\sum_{i=1}^{2M}a_{i}h_{i}\left(\xi \right)$$

in which $a_{i}$(i=1,…,2M) represent wavelet coefficients to be determined.

Implementation of the HWDM for solving differential equations requires computation of successive integrals of the basis function defined in Equation (18). These integrals can be derived analytically as follows [49]:

(20) $$p_{n,i}\left(\xi \right)=\frac{\xi^{n}}{n!},\;\mathrm{for}\;i=1;$$

(21) $$p_{n,i}\left(\xi \right)=\left\{\begin{array}{l} 0,\;\xi \leq \xi_{1}(i)\\ \frac{1}{n!}{\left(\xi -\xi_{1}(i)\right)}^{n},\;\xi_{1}(i)<\xi \leq \xi_{2}(i)\\ \frac{1}{n!}\left[{\left(\xi -\xi_{1}(i)\right)}^{n}-2{\left(\xi -\xi_{2}(i)\right)}^{n}\right],\;\xi_{2}(i)<\xi \leq \xi_{3}(i)\\ \frac{1}{n!}\left[{\left(\xi -\xi_{1}(i)\right)}^{n}-2{\left(\xi -\xi_{2}(i)\right)}^{n}+{\left(\xi -\xi_{3}(i)\right)}^{n}\right],\;\xi >\xi_{3}(i) \end{array}\right.,\;\mathrm{for}\;i>1.$$

where n denotes the integration order of the Haar wavelet function. For n=0, $h_{i}\left(\xi \right)$ is achieved.

Consider a uniform partition of the interval [0, 1] into 2M subintervals of equal length Δξ=1/2M; the collocation points are located at:

(22) $$\xi_{l}=\frac{\left(l-0.5\right)}{2M},\;l=1,\;2,\;\ldots ,\;2M$$

By evaluating Equations (18), (20), and (21) at the collocation points specified in Equation (22), we construct the Haar coefficient matrix H and its corresponding integral operator matrices $P^{\left(n\right)}$ as follows:

(23) $$H(i,l)=h_{i}(\xi_{l}),\;P^{\left(n\right)}(i,l)=p_{n,i}\left(\xi_{l}\right)$$

where H and $P^{\left(n\right)}$ are 2M×2M matrices and will be used to discretize the given governing equations in the next subsection.

### 3.2. Implementation of the HWDM

Firstly, in order to apply the HWDM to discretize the governing equations of the conical shell segment, the displacement field should be converted to the unit interval [0,1]. Thus, the following non-dimensional variable $\xi_{c}$ is introduced.

(24) $$\xi_{c}=x_{c}/L_{c}$$

Substitution of Equation (24) into Equation (13) yields the governing equations dependent on a single variable $\xi_{c}$:

(25) $$\begin{array}{l} {\tilde{G}}_{111}U^{c}+{\tilde{G}}_{112}\frac{dU^{c}}{d\xi_{c}}+{\tilde{G}}_{113}\frac{d^{2}U^{c}}{d\xi_{c}^{2}}+{\tilde{G}}_{121}V^{c}+{\tilde{G}}_{122}\frac{dV^{c}}{d\xi_{c}}+{\tilde{G}}_{131}W^{c}+{\tilde{G}}_{132}\frac{dW^{c}}{d\xi_{c}}+{\tilde{G}}_{141}\Phi_{x}^{c}\\ +{\tilde{G}}_{142}\frac{d\Phi_{x}^{c}}{d\xi_{c}}+{\tilde{G}}_{143}\frac{d^{2}\Phi_{x}^{c}}{d\xi_{c}^{2}}+{\tilde{G}}_{151}\Phi_{\theta }^{c}+{\tilde{G}}_{152}\frac{d\Phi_{\theta }^{c}}{d\xi_{c}}+I_{0}\omega^{2}R^{2}U^{c}+I_{1}\omega^{2}R^{2}\Phi_{x}^{c}=0\\ {\tilde{G}}_{211}U^{c}+{\tilde{G}}_{212}\frac{dU^{c}}{d\xi_{c}}+{\tilde{G}}_{221}V^{c}+{\tilde{G}}_{222}\frac{dV^{c}}{d\xi_{c}}+{\tilde{G}}_{223}\frac{d^{2}V^{c}}{d\xi_{c}^{2}}+{\tilde{G}}_{231}W^{c}+{\tilde{G}}_{241}\Phi_{x}^{c}+{\tilde{G}}_{242}\frac{d\Phi_{x}^{c}}{d\xi_{c}}\\ +{\tilde{G}}_{251}\Phi_{\theta }^{c}+{\tilde{G}}_{252}\frac{d\Phi_{\theta }^{c}}{d\xi_{c}}+{\tilde{G}}_{253}\frac{d^{2}\Phi_{\theta }^{c}}{d\xi_{c}^{2}}+I_{0}\omega^{2}R^{2}V^{c}+I_{1}\omega^{2}R^{2}\Phi_{\theta }^{c}=0\\ {\tilde{G}}_{311}U^{c}+{\tilde{G}}_{312}\frac{dU^{c}}{d\xi_{c}}+{\tilde{G}}_{321}V^{c}+{\tilde{G}}_{331}W^{c}+{\tilde{G}}_{332}\frac{dW^{c}}{d\xi_{c}}+{\tilde{G}}_{333}\frac{d^{2}W^{c}}{d\xi_{c}^{2}}+{\tilde{G}}_{341}\Phi_{x}^{c}+{\tilde{G}}_{342}\frac{d\Phi_{x}^{c}}{d\xi_{c}}\\ +{\tilde{G}}_{351}\Phi_{\theta }^{c}+I_{0}\omega^{2}R^{2}W^{c}=0\\ {\tilde{G}}_{411}U^{c}+{\tilde{G}}_{412}\frac{dU^{c}}{d\xi_{c}}+{\tilde{G}}_{413}\frac{d^{2}U^{c}}{d\xi_{c}^{2}}+{\tilde{G}}_{421}V^{c}+{\tilde{G}}_{422}\frac{dV^{c}}{d\xi_{c}}+{\tilde{G}}_{431}W^{c}+{\tilde{G}}_{432}\frac{dW^{c}}{d\xi_{c}}+{\tilde{G}}_{441}\Phi_{x}^{c}\\ +{\tilde{G}}_{442}\frac{d\Phi_{x}^{c}}{d\xi_{c}}+{\tilde{G}}_{443}\frac{d^{2}\Phi_{x}^{c}}{d\xi_{c}^{2}}+{\tilde{G}}_{451}\Phi_{\theta }^{c}+{\tilde{G}}_{452}\frac{d\Phi_{\theta }^{c}}{d\xi_{c}}+I_{1}\omega^{2}R^{2}U^{c}+I_{2}\omega^{2}R^{2}\Phi_{x}^{c}=0\\ {\tilde{G}}_{511}U^{c}+{\tilde{G}}_{512}\frac{dU^{c}}{d\xi_{c}}+{\tilde{G}}_{521}V^{c}+{\tilde{G}}_{522}\frac{dV^{c}}{d\xi_{c}}+{\tilde{G}}_{523}\frac{d^{2}V^{c}}{d\xi_{c}^{2}}+{\tilde{G}}_{531}W^{c}+{\tilde{G}}_{541}\Phi_{x}^{c}+{\tilde{G}}_{542}\frac{d\Phi_{x}^{c}}{d\xi_{c}}\\ +{\tilde{G}}_{551}\Phi_{\theta }^{c}+{\tilde{G}}_{552}\frac{d\Phi_{\theta }^{c}}{d\xi_{c}}+{\tilde{G}}_{553}\frac{d^{2}\Phi_{\theta }^{c}}{d\xi_{c}^{2}}+I_{1}\omega^{2}R^{2}U^{c}+I_{2}\omega^{2}R^{2}\Phi_{x}^{c}=0 \end{array}$$

where ${\tilde{G}}_{ijk}$(i,j=1~5, k=1~3) denote the constants given in Appendix A.

Following the HWDM framework, the highest-order derivatives of the displacement functions with respect to the normalized coordinate $\xi_{c}$ are represented through truncated Haar series expansions. Equation (25) reveals that all displacement and rotation components exhibit at most second-order derivatives. Consequently, the corresponding highest derivatives of all the components are approximated by:

(26) $$\frac{d^{2}}{d\xi_{c}^{2}}\left\{U^{c}(\xi_{c}),V^{c}(\xi_{c}),W^{c}(\xi_{c}),\Phi_{x}^{c}(\xi_{c}),\Phi_{\theta }^{c}(\xi_{c})\right\}=\sum_{i=1}^{2M}\left\{a_{i},b_{i},c_{i},d_{i},e_{i}\right\}h_{i}(\xi_{c})$$

where $a_{i}$, $b_{i}$, $c_{i}$, $d_{i}$, and $e_{i}$ denote the Haar wavelet coefficients, while $h_{i}(\xi_{c})$ indicates the Haar wavelet function defined in Equation (18). The truncated number 2M is equal to $2^{J+1}$. Using the property of the integrals of the Haar wavelet function and integrating Equation (26) leads to the following expressions:

(27) $$\begin{array}{l} \frac{d}{d\xi_{c}}\left\{U^{c}(\xi_{c}),V^{c}(\xi_{c}),W^{c}(\xi_{c}),\Phi_{x}^{c}(\xi_{c}),\Phi_{\theta }^{c}(\xi_{c})\right\}=\sum_{i=1}^{2M}\left\{a_{i},b_{i},c_{i},d_{i},e_{i}\right\}p_{1,i}(\xi_{c})\\ +\frac{d}{d\xi_{c}}\left\{U^{c}(0),V^{c}(0),W^{c}(0),\Phi_{x}^{c}(0),\Phi_{\theta }^{c}(0)\right\}\\ \left\{U^{c}(\xi_{c}),V^{c}(\xi_{c}),W^{c}(\xi_{c}),\Phi_{x}^{c}(\xi_{c}),\Phi_{\theta }^{c}(\xi_{c})\right\}=\sum_{i=1}^{2M}\left\{a_{i},b_{i},c_{i},d_{i},e_{i}\right\}p_{2,i}(\xi_{c})\\ +\xi_{c}\frac{d}{d\xi_{c}}\left\{U^{c}(0),V^{c}(0),W^{c}(0),\Phi_{x}^{c}(0),\Phi_{\theta }^{c}(0)\right\}+\left\{U^{c}(0),V^{c}(0),W^{c}(0),\Phi_{x}^{c}(0),\Phi_{\theta }^{c}(0)\right\} \end{array}$$

where $d\left\{U^{c}(0),V^{c}(0),W^{c}(0),\Phi_{x}^{c}(0),\Phi_{\theta }^{c}(0)\right\}/d\xi_{c}$ and $\left\{U^{c}(0),V^{c}(0),W^{c}(0),\Phi_{x}^{c}(0),\Phi_{\theta }^{c}(0)\right\}$ are the integration constants to be determined. It should be noted here that for each displacement components, the number of the coefficients to be determined is 2M+2.

Consider a uniform partition of the interval [0, 1] into 2M subintervals of equal length Δξ=1/2M; the collocation points are located at:

(28) $$\xi_{k}=\frac{\left(k-0.5\right)}{2M},\;k=1,\;2,\;\ldots ,\;2M$$

Evaluating Equations (26) and (27) at the specified collocation points yields the following matrix representation:

(29) $$\begin{array}{l} \frac{d^{2}U^{c}}{d\xi_{c}^{2}}=Ha,\;\frac{d^{2}V^{c}}{d\xi_{c}^{2}}=Hb,\;\frac{d^{2}W^{c}}{d\xi_{c}^{2}}=Hc,\;\frac{d^{2}\Phi_{x}^{c}}{d\xi_{c}^{2}}=Hd,\;\frac{d^{2}\Phi_{\theta }^{c}}{d\xi_{c}^{2}}=He,\\ \frac{dU^{c}}{d\xi_{c}}=P^{\left(1\right)}a+Q_{1}F,\;\frac{dV^{c}}{d\xi_{c}}=P^{\left(1\right)}b+Q_{1}G,\;\frac{dW^{c}}{d\xi_{c}}=P^{\left(1\right)}c+Q_{1}T,\\ \frac{d\Phi_{x}^{c}}{d\xi_{c}}=P^{\left(1\right)}d+Q_{1}K,\;\frac{d\Phi_{\theta }^{c}}{d\xi_{c}}=P^{\left(1\right)}e+Q_{1}L,\\ U^{c}=P^{(2)}a+Q_{2}F,\;V^{c}=P^{(2)}b+Q_{2}G,\;W^{c}=P^{(2)}c+Q_{2}T,\\ \Phi_{x}^{c}=P^{(2)}d+Q_{2}K,\;\Phi_{\theta }^{c}=P^{(2)}e+Q_{2}L \end{array}$$

where $a={\left[\begin{array}{cccc} a_{1} & a_{2} & \ldots & a_{2M} \end{array}\right]}^{T}$, $b={\left[\begin{array}{cccc} b_{1} & b_{2} & \ldots & b_{2M} \end{array}\right]}^{T}$, $c={\left[\begin{array}{cccc} c_{1} & c_{2} & \ldots & c_{2M} \end{array}\right]}^{T}$, $d={\left[\begin{array}{cccc} d_{1} & d_{2} & \ldots & d_{2M} \end{array}\right]}^{T}$, $e={\left[\begin{array}{cccc} e_{1} & e_{2} & \ldots & e_{2M} \end{array}\right]}^{T}$ and $F={\left[\begin{array}{cc} dU^{c}(0)/d\xi_{c} & U^{c}(0) \end{array}\right]}^{T}$, $G={\left[\begin{array}{cc} dV^{c}(0)/d\xi_{c} & V^{c}(0) \end{array}\right]}^{T}$, $T={\left[\begin{array}{cc} dW^{c}(0)/d\xi_{c} & W^{c}(0) \end{array}\right]}^{T}$, $K={\left[\begin{array}{cc} d\Phi_{x}^{c}(0)/d\xi_{c} & \Phi_{x}^{c}(0) \end{array}\right]}^{T}$, $L={\left[\begin{array}{cc} d\Phi_{\theta }^{c}(0)/d\xi_{c} & \Phi_{\theta }^{c}(0) \end{array}\right]}^{T}$. Here, H, $P^{(1)}$ and $P^{(2)}$ represent 2M×2M matrices defined in Equation (23). $Q_{1}$ and $Q_{2}$ are constant matrices as follows:

(30) $$Q_{1}={\left[\begin{array}{cccc} 1 & 1 & \ldots & 1\\ 0 & 0 & \ldots & 0 \end{array}\right]}_{2\times 2M}^{T},\;Q_{2}={\left[\begin{array}{cccc} \xi_{1} & \xi_{2} & \ldots & \xi_{2M}\\ 1 & 1 & \ldots & 1 \end{array}\right]}^{T}$$

Through this formulation, all displacement and rotation components, along with their derivatives, are systematically expressed in the form of Haar wavelet series and their integrals.

In the following, we will employ the HWDM to discretize the motion equations of the conical segment. Substituting Equation (29) into Equation (25) transforms the governing equations into algebraic equations with respect to the unknown wavelet coefficients. To illustrate this procedure, we specifically consider the third equation of Equation (25). Through substitution and algebraic manipulation, we obtain a system of linear algebraic equations expressed as:

(31) $${\left[\begin{array}{c} {\tilde{G}}_{311}P^{(2)}+{\tilde{G}}_{312}P^{\left(1\right)}\\ {\tilde{G}}_{321}P^{(2)}\\ {\tilde{G}}_{331}P^{(2)}+{\tilde{G}}_{332}P^{\left(1\right)}+{\tilde{G}}_{333}H\\ {\tilde{G}}_{341}P^{(2)}+{\tilde{G}}_{342}P^{\left(1\right)}\\ {\tilde{G}}_{351}P^{(2)} \end{array}\right]}^{T}X_{d}^{c}+{\left[\begin{array}{c} {\tilde{G}}_{311}Q_{2}+{\tilde{G}}_{312}Q_{1}\\ {\tilde{G}}_{321}Q_{2}\\ {\tilde{G}}_{331}Q_{2}+{\tilde{G}}_{332}Q_{1}\\ {\tilde{G}}_{341}Q_{2}+{\tilde{G}}_{342}Q_{1}\\ {\tilde{G}}_{351}Q_{2} \end{array}\right]}^{T}X_{b}^{c}=-\omega^{2}\left({\left[\begin{array}{c} 0\\ 0\\ I_{0}R^{2}P^{(2)}\\ 0\\ 0 \end{array}\right]}^{T}X_{d}^{c}+{\left[\begin{array}{c} 0\\ 0\\ I_{0}R^{2}Q_{2}\\ 0\\ 0 \end{array}\right]}^{T}X_{b}^{c}\right)$$

in which $X_{d}^{c}={\left[\begin{array}{ccccc} a & b & c & d & e \end{array}\right]}^{T}$, while $X_{b}^{c}={\left[\begin{array}{ccccc} F & G & T & K & L \end{array}\right]}^{T}$. The superscript c denotes the conical shell segment.

Using the identical discretization procedure to the remaining four equations in Equation (25), we obtain the complete discretized governing equation system for the conical shell segment. Through global matrix assembly, this yields the following system of linear algebraic equations:

(32) $$K_{dd}^{c}X_{d}^{c}+K_{db}^{c}X_{b}^{c}=\omega^{2}\left(M_{dd}^{c}X_{d}^{c}+M_{db}^{c}X_{b}^{c}\right)$$

where $M_{dd}^{c}$, $M_{db}^{c}$, $K_{dd}^{c}$, and $K_{db}^{c}$ denote the corresponding mass and stiffness matrices of the conical shell segment.

Using the same approach and setting α to be zero, the discretized governing equations of the cylindrical shell segment are obtained as follows:

(33) $$K_{dd}^{l}X_{d}^{l}+K_{db}^{l}X_{b}^{l}=\omega^{2}\left(M_{dd}^{l}X_{d}^{l}+M_{db}^{l}X_{b}^{l}\right)$$

where $M_{dd}^{l}$, $M_{db}^{l}$, $K_{dd}^{l}$, and $K_{db}^{l}$ denote the corresponding mass and stiffness matrices of the cylindrical shell. $X_{d}^{l}={\left[\begin{array}{ccccc} a^{l} & b^{l} & c^{l} & d^{l} & e^{l} \end{array}\right]}^{T}$ and $X_{b}^{l}={\left[\begin{array}{ccccc} F^{l} & G^{l} & T^{l} & K^{l} & L^{l} \end{array}\right]}^{T}$ are the unknown wavelet coefficients and integration constant of the cylindrical shell segment.

The discretization of the continuity condition at the junction and the boundary condition at the two ends is carried out in the same way. First, discretization of the continuity condition Equation (14) yields the following:

(34) $$K_{bd}^{jc}X_{d}^{c}+K_{bb}^{jc}X_{b}^{c}+K_{bd}^{jl}X_{d}^{l}+K_{bb}^{jl}X_{b}^{l}=0$$

where $K_{bd}^{jc}$, $K_{bb}^{jc}$, $K_{bd}^{jl}$ and $K_{bb}^{jl}$ are the coupled stiffness matrices at the junction of the CCCSs. Similarly, the boundary conditions on both ends of the CCCSs are discretized, and then the following expressions are obtained:

(35) $$K_{bd}^{c}X_{d}^{c}+K_{bb}^{c}X_{b}^{c}=0,\;K_{bd}^{l}X_{d}^{l}+K_{bb}^{l}X_{b}^{l}=0$$

where $K_{bd}^{c}$ and $K_{bb}^{c}$ are the boundary stiffness matrices at the conical end, and $K_{bd}^{l}$ and $K_{bb}^{l}$ are the corresponding matrices at the cylindrical end.

Assembling Equations (32)–(35) results in the global algebraic equations for the free vibration of FG-CNTRC CCCSs interacting with the elastic foundation as follows:

(36) $$\left(\left[\begin{array}{cccc} K_{dd}^{c} & K_{db}^{c} & 0 & 0\\ K_{bd}^{jc} & K_{bb}^{jc} & K_{bd}^{jl} & K_{bb}^{jl}\\ 0 & 0 & K_{db}^{l} & K_{db}^{l}\\ K_{bd}^{c} & K_{bb}^{c} & 0 & 0\\ 0 & 0 & K_{bd}^{l} & K_{bb}^{l} \end{array}\right]-\omega^{2}\left[\begin{array}{cccc} M_{dd}^{c} & M_{db}^{c} & 0 & 0\\ 0 & 0 & 0 & 0\\ 0 & 0 & M_{dd}^{l} & M_{db}^{l}\\ 0 & 0 & 0 & 0\\ 0 & 0 & 0 & 0 \end{array}\right]\right)\left[\begin{array}{c} X_{d}^{c}\\ X_{b}^{c}\\ X_{d}^{l}\\ X_{b}^{l} \end{array}\right]=0$$

The natural frequencies of the FG-CNTRC CCCS considered here are achieved by evaluating the eigenvalue problem in Equation (36). Meanwhile, the mode shapes for the given circumferential wave number can be determined by substituting the obtained wavelet coefficients and integration constants into the displacement expressions.

## 4. Results and Discussion

Free vibration analysis of the FG-CNTRC CCCSs supported by an elastic foundation is presented in this section to verify the validity and accuracy of the proposed method. Firstly, the convergence and comparison studies are conducted by comparing the obtained results with both the existing literature results and FEM solutions. Afterwards, detailed parametric investigations are carried out to examine the effects of the distribution type and volume fraction of CNTs, boundary condition, geometric parameters, and the stiffness coefficients of the foundation on vibration behaviors of the FG-CNTRC CCCSs.

Unless otherwise specified, the CNTRC adopted here consists of a polymethyl methacrylate (PMMA) matrix reinforced by (10, 10) armchair single-walled CNTs. The mechanical properties of the PMMA matrix are given as follows: $E^{m}=2.5\;\mathrm{GPa}$, $\rho^{m}=1150\;\mathrm{kg}/m^{3}$, $v^{m}=0.34$. The corresponding properties of the CNTs are summarized in Table 1. As stated in Equation (4), three efficiency parameters are incorporated to account for size-dependent material behavior of the CNTRC. For three diverse $V_{CNT}^{*}$, these parameters are taken as: $\eta_{1}$ = 0.137 and $\eta_{2}$ = 1.022 for $V_{CNT}^{*}$ = 0.12; $\eta_{1}$ = 0.142 and $\eta_{2}$ = 1.626 for $V_{CNT}^{*}$ = 0.17; and $\eta_{1}$ = 0.141 and $\eta_{2}$ = 1.585 for $V_{CNT}^{*}$ = 0.28. For each case, $\eta_{3}$ is taken as $0.7\eta_{2}$.

In this study, three diverse boundary conditions (i.e., C, SD, F) are considered. A letter string is adopted to denote the boundary conditions on both ends of the CCCS. For instance, C-F represents the CCCS has the fully clamped boundary condition at the conical end and the free one at the cylindrical end. The dimensionless stiffness coefficients of the Pasternak-type foundation are expressed as:

(37) $$K_{w}=k_{w}R_{2}^{4}/D_{m},\;K_{s}=k_{s}R_{2}^{2}/D_{m}$$

where $D_{m}=E^{m}h^{3}/\left[12\left(1-v^{m}v^{m}\right)\right]$. Unless otherwise specified, the geometric parameters of the CCCS considered here are taken as: $R_{1}=0.4226\;m$, $R_{2}=L_{l}=1\;m$, h=0.01 m, and $\alpha =30^{\circ }$.

### 4.1. Convergence and Comparison Studies

This subsection first investigates the convergence characteristics of the proposed HWDM for the vibration response of FG-CNTRC CCCSs and the maximal level of resolution J is determined for the truncated Haar series to ensure numerical accuracy. Furthermore, to evaluate the accuracy of the HWDM, comparison studies with the other available solutions are performed in the limit cases. In what follows, the non-dimensional natural frequencies are expressed as:

(38) $$\Omega_{nm}=\omega_{nm}R_{2}\sqrt{\rho^{m}\left(1-\nu^{m}\nu^{m}\right)/E^{m}}$$

where the subscripts m and n denote the meridional and circumferential wave number of the CCCS.

Table 2 displays the non-dimensional frequencies $\Omega_{nm}$ of FG-X CCCSs under the C-C boundary condition with different J. The foundation stiffness coefficients are $K_{w}=K_{s}=0$ and $V_{CNT}^{*}$ is 0.12. The results demonstrate stable convergence with increasing J. The estimated numerical order of convergence, defined as:

(39) $$k_{i}=\log\left(\frac{R_{i-2}-R_{i-1}}{R_{i-1}-R_{i}}\right)/\log(2)$$

where $R_{i}$ denotes the corresponding result for J=i, are also included in Table 2. It is seen from Table 2 that the numerical convergence order approaches 2, which agrees with the theoretical convergence rate of HWDM established in Ref. [50]. Furthermore, the results become essentially invariant when J reaches a critical value J=7. Therefore, the optimal resolution level J=7 is adopted in the subsequent cases.

The following case presents a comparative study of the homogeneous isotropic CCCSs supported by elastic foundations with diverse boundary conditions. The first five natural frequencies are calculated and compared with FEM results, as summarized in Table 3. The material parameters of the joined structures are as follows: E=211 GPa, v=0.3 and $\rho =7800\;\mathrm{kg}/m^{3}$. In the FEM analysis (using ANSYS 2023 R1), the conical shell and cylindrical shell are discretized with SHELL63 elements using mesh sizes of 120 × 40 and 120 × 30, respectively. Three boundary conditions, i.e., C-C, C-F, and SD-SD, are studied in this example. As shown in Table 3, the present results for different boundary conditions and elastic foundation parameters agree well with the FEM solutions. The discrepancy between the present and the FEM solutions is very small and is limited to 0.14% for the worst case. Furthermore, the computational efficiency of the proposed method is compared with that of the FEM program. The computational time of HWDM implemented in the mathematical software MATLAB R2024a is about 9 s, and for the FEM, it is about 40 s. Consequently, the proposed methodology demonstrates significantly higher computational efficiency compared to the FEM.

In the next example, the non-dimensional frequencies $\Omega_{nm}$ of the F-C homogeneous isotropic CCCSs are given in Table 4, comparing them with both literature results and FEM solutions. The geometric parameters and material properties of the shell used here are consistent with those in the previous example. The elastic foundation is not considered. Table 4 demonstrates excellent agreement between the present results and the published literature values [44,51,52] as well as the FEM results. The maximum discrepancies between the present and literature values are 0.31% for Ref. [44], 0.03% for Ref. [51], and 0.02% for Ref. [52]. For FEM, the maximum discrepancy reaches 0.33%.

Convergence analysis and comparative studies demonstrate that the proposed HWDM exhibits stable second-order convergence, high computational efficiency, and excellent accuracy in analyzing the free vibration of CCCSs on elastic foundations

### 4.2. Free Vibration of FG-CNTRC CCCSs Resting on Elastic Foundations

This subsection investigates the free vibration characteristics of FG-CNTRC CCCSs on elastic foundations, adopting the proposed HWDM. Systematic analyses are conducted to examine the influences of key parameters, including CNT volume fraction, CNT distribution patterns, boundary conditions, foundation stiffness coefficients, and geometric dimensions on the shell’s natural frequencies.

#### 4.2.1. Effects of Material Parameters and Boundary Conditions on Natural Frequencies

As the first case, the effects of the CNT volume fraction and CNT distribution patterns on the natural frequencies of the CCCSs on the elastic foundation are examined. Table 5 presents the first four non-dimensional frequencies $\Omega_{i}(i=1\sim 4)$ of FG-CNTRC CCCSs with and without the elastic foundation. Three volume fractions of CNTs and five distribution types are considered in this case. Here, the geometric parameters are as follows: $\alpha =45^{\circ }$, $L_{c}=L_{l}=R_{2}=1\;m$, and $h=0.01R_{2}$, and the boundary condition is set to SD-SD. It is found from Table 5 that the frequencies exhibit a positive correlation with the CNT volume fraction, primarily attributed to the enhanced structural stiffness induced by CNT reinforcement. Among the five CNT distribution patterns, the FG-X type yields the highest frequencies, whereas the FG-O type produces the lowest. This phenomenon may be explained by the superior efficiency of reinforcements concentrated near the top and bottom surfaces in enhancing shell stiffness compared to those distributed around the mid-surface. Additionally, the presence of an elastic foundation significantly influences the natural frequencies of the CCCS.

The effect of boundary conditions on the vibration behaviors of the CNTRC CCCSs resting on elastic foundations is investigated in this case. Three classical boundary conditions (i.e., C, F, SD) and their combinations are investigated. Table 6 lists the fundamental frequency $\Omega_{1}$ for FG-X CCCSs resting on elastic foundations under various boundary conditions. The geometric sizes are consistent with those used in the previous case. The total volume fraction $V_{CNT}^{*}$ is taken as 0.17. The fundamental frequencies with various foundation coefficients are also covered in Table 6. As expected, among all the cases, the CCCS with C-C boundary condition exhibits the highest fundamental frequency, followed by SD-C, C-SD, SD-SD, F-C, C-F, SD-F, and F-SD. This can be explained by the fact that the more degrees of freedom that are restrained, the greater the stiffness of the shell. Moreover, it is found from Table 6 that an increase in the value of $K_{w}$ or $K_{s}$ yields higher fundamental frequencies.

#### 4.2.2. Effects of Foundation Stiffnesses on the Natural Frequencies

**Figure 2:**
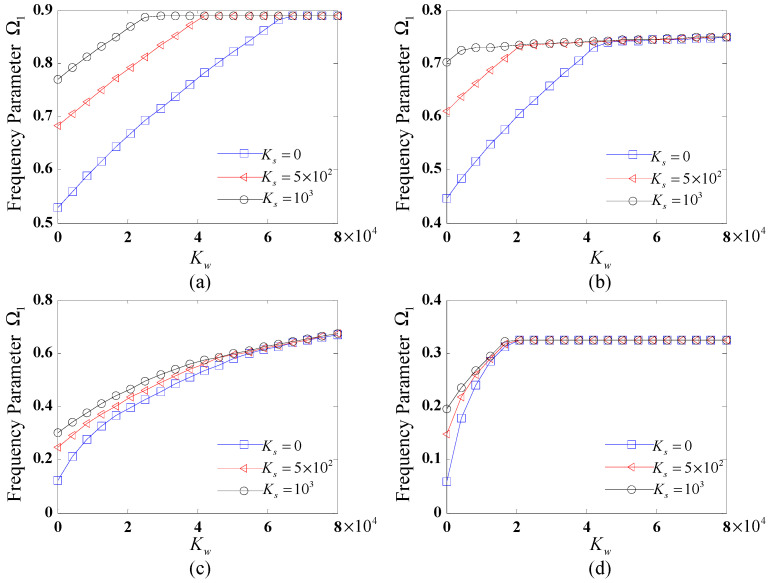
Variation of fundamental frequency $\Omega_{1}$ with the Winkler coefficient for different shear coefficients subjected to distinct boundary conditions: (**a**) C-C; (**b**) SD-SD; (**c**) F-C; (**d**) C-F.

**Figure 3:**
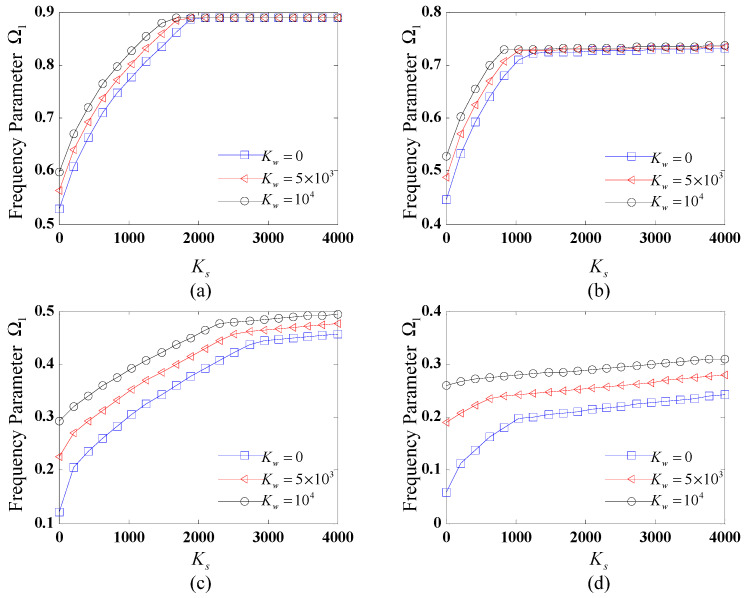
Variation of fundamental frequency $\Omega_{1}$ with the shear coefficient for different Winkler coefficients subjected to distinct boundary conditions: (**a**) C-C; (**b**) SD-SD; (**c**) F-C; (**d**) C-F.

To systematically examine the influence of elastic foundation stiffness on the vibrational characteristics of FG-CNTRC CCCSs, Figure 2 and Figure 3 present the variation of the fundamental frequency $\Omega_{1}$ with respect to both Winkler and shear coefficients. Four distinct boundary conditions, i.e., C-C, SD-SD, F-C, and C-F, are considered. The geometric parameters and CNTRC material properties remain consistent with the previous case study.

Figure 2 illustrates the variation of the fundamental frequency $\Omega_{1}$ with the Winkler coefficient for different shear coefficients ($K_{s}$ = 0, 500 and 1000) under distinct boundary conditions. The results reveal that the fundamental frequency initially increases rapidly with the Winkler coefficient, reaches a maximum, and then stabilizes. For small $K_{w}$, the fundamental frequency rises with increasing $K_{s}$. With the increase in $K_{w}$ to a certain value, it becomes insensitive and remains unchanged for different values of $K_{s}$. Moreover, the boundary conditions significantly influence the growth rate and trend of the fundamental frequency parameters with $K_{w}$. Among the tested boundary conditions, the C-C case yields the highest fundamental frequencies, followed by SD-SD, F-C, and C-F.

The variations of the fundamental frequency $\Omega_{1}$ with the shear coefficients for different values of the Winkler coefficient, subjected to distinct boundary conditions, are shown in Figure 3. The values of the Winkler coefficients considered are $K_{w}$ = 0, 5000, and 10,000. It can be seen from Figure 3 that the fundamental frequency initially increases rapidly with the shear coefficient of the elastic foundation, reaches a maximum, and then stabilizes for different boundary conditions. For SD-SD and C-C boundary conditions, $\Omega_{1}$ reaches a maximum value and then remains stable with the increase in $K_{s}$. For F-C and C-F ones, $\Omega_{1}$ first increases rapidly and then rises slowly with the increase in $K_{s}$. Furthermore, it is observed that the increase in the value of $K_{w}$ results in the increased fundamental frequency for C-F and F-C boundary conditions. For the SD-SD and C-C ones, these trends become different: For a small value of $K_{s}$, the increase in $K_{w}$ results in an increased $\Omega_{1}$ and with an increase in $K_{s}$ to a certain value, $\Omega_{1}$ stays unchanged for different values of $K_{w}$.

From Figure 2 and Figure 3, it can be inferred that the increase in the values of foundation stiffness coefficients within a certain range results in the increased fundamental frequency of the CCCS, and when the values of stiffness coefficient reach a certain value, the influence of the foundation coefficient on the natural frequency becomes insensitive. The above certain range or value is strongly influenced by the specific boundary conditions applied.

#### 4.2.3. Effects of Geometric Parameter on Natural Frequencies

In the following, we conduct some numerical cases to study the effects of the geometric configurations, covering the length-to-radius ratio $L_{l}/R_{2}$, the thickness-to-radius ratio $h/R_{2}$, and the semi-vertex angle α on the fundamental frequencies of FG-CNTRC CCCSs supported by an elastic foundation.

Table 7 summarizes the fundamental frequencies for FG-X CNTRC CCCSs supported by an elastic foundation for different $L_{l}/R_{2}$ and $h/R_{2}$ under various boundary conditions. The $V_{CNT}^{*}$ is taken as 0.17. The geometric sizes of the CCCS considered are $L_{c}=R_{2}=1\;m$ and $\alpha =45^{\circ }$. Three distinct boundary conditions are considered: C-C, SD-SD, and F-C. From Table 7, it is found that the fundamental frequencies decrease gradually with the increase in $L_{l}/R_{2}$ for all the given $h/R_{2}$ and boundary conditions. This phenomenon may be explained by the fact that an increase in the length-to-radius ratio yields a decrease in the shell’s stiffness. Moreover, the results reveal an increase in $h/R_{2}$ results in an increase in fundamental frequencies. This phenomenon can be attributed to the enhanced structural stiffness resulting from increased shell thickness, which consequently elevates the fundamental vibration frequencies.

**Figure 4:**
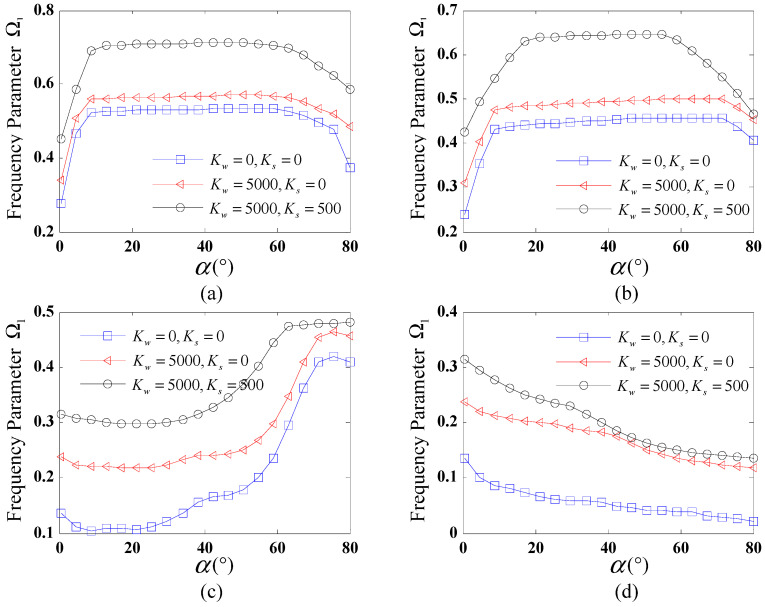
Variation of fundamental frequencies $\Omega_{1}$ with semi-vertex angle α for different foundation coefficients under various boundary conditions: (**a**) C-C; (**b**) SD-SD; (**c**) F-C; (**d**) C-F.

Figure 4 shows the variation of the fundamental frequency $\Omega_{1}$ for FG-X CCCSs resting on an elastic foundation against the semi-vertex angle α. Four distinct boundary conditions are considered: C-C, SD-SD, C-F, and F-C. The given geometric parameters of the shell are $L_{c}=L_{l}=R_{2}=1\;m$ and $h=0.01R_{2}$, and the $V_{CNT}^{*}$ is taken as 0.17. The range of α is taken from $0^{\circ }$ to $80^{\circ }$. Three sets of foundation stiffness coefficients ($K_{w}$, $K_{s}$) are used here: (0, 0), (5000, 0), and (5000, 500). As shown in Figure 4, different trends of the fundamental frequency against α are observed for various boundary conditions. For C-C and SD-SD boundary conditions, as shown in Figure 4a,b, the fundamental frequencies first increase rapidly and then remain constant with the increase in α; when α reaches a critical value, the fundamental frequencies gradually decrease. For the F-C boundary condition, a significantly fluctuating trend is observed from Figure 4c, demonstrating a complex relationship between the fundamental frequency and semi-vertex angle. It is observed from Figure 4d that for the C-F boundary condition, the frequency of the CCCS monotonically decreases with the rise of α. In addition, it is evident that under the given boundary condition, the trends of the fundamental frequency against α nearly coincide for different foundation stiffness coefficients.

#### 4.2.4. Mode Shapes of FG-CNTRC CCCSs Supported by an Elastic Foundation

**Figure 5:**
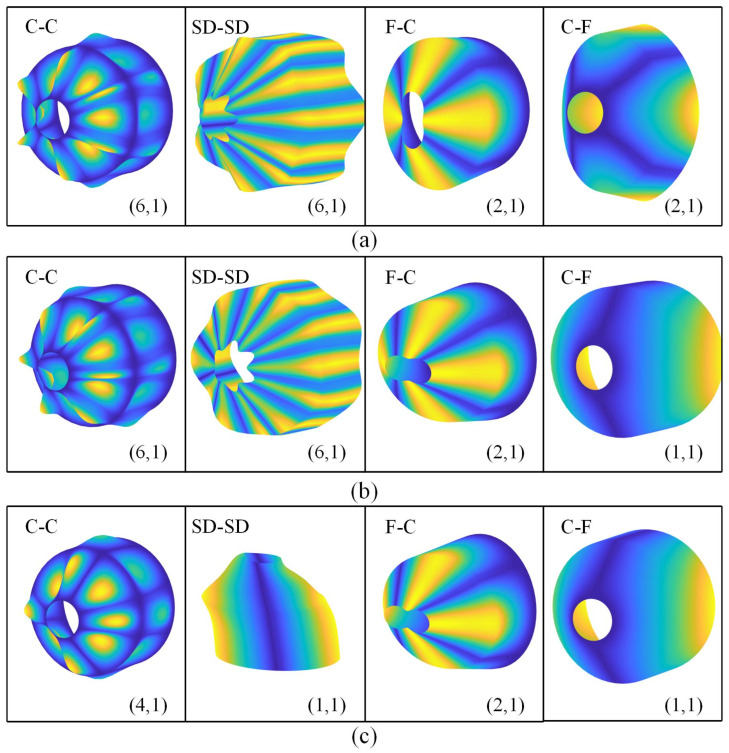
Mode shapes corresponding to the fundamental frequencies of FG-X CCCSs for different foundation coefficients under various boundary conditions: (**a**) $K_{w}=0$, $K_{s}=0$; (**b**) $K_{w}=10^{4}$, $K_{s}=0$; (**c**) $K_{w}=10^{4}$, $K_{s}=10^{3}$.

In addition to determining the natural frequencies, the proposed methodology enables efficient computation of mode shapes for the CCCS. Figure 5 illustrates the mode shapes related to the fundamental frequencies of the CCCS resting on elastic foundations under various boundary conditions. Here, $V_{CNT}^{*}$ is taken as 0.17 and the distribution type is FG-X. The given geometric sizes of the CCCS are: $L_{c}=L_{l}=R_{2}=1\;m$, $h=0.01R_{2}$, and $\alpha =45^{\circ }$. Four distinct boundary conditions are adopted: C-C, SD-SD, C-F, and F-C. Three sets of foundation coefficients are used here: (0, 0) for no elastic foundation, ($10^{4}$, 0) for Winkler-type foundation and ($10^{4}$, $10^{3}$) for Pasternak-type foundation. The circumferential and meridional wave number (n, m) for the corresponding mode shape are also shown in Figure 5.

It is observed from Figure 5 that the vibration of each shell segment is coupled and the deformation at the junction of the CCCS is continuous. For various boundary conditions, the mode shapes are quite different. In addition, it is found that for the given boundary condition, the mode shapes of the joined shell may be changed as the foundation stiffness coefficients vary. In other words, the appearance of the elastic foundation affects the mode shapes of the joined shell, which is determined by the foundation stiffness coefficients.

Hereto, the free vibration behaviors of FG-CNTRC CCCS were successfully treated using the HWDM. While Majak et al. [36] established the foundational calculation framework of the HWDM for the vibration analysis of elementary plate and shell configurations, our work uniquely addresses the vibration behaviors of CCCSs by the discretization and assembling of each shell segment along with the displacement and force continuous conditions via the HWDM, which is the methodological innovation of this work. Moreover, the proposed numerical framework can be straightforwardly applied to other joined shells, e.g., conical–cylindrical–conical or conical–conical shells. It is noteworthy that the present methodology is exclusively applicable to linear free-vibration analysis of CCCSs, while geometrically nonlinear or large-displacement vibration behaviors require future extensions, which are beyond the scope of the current methodology.

## 5. Conclusions

The free vibration behaviors of FG-CNTRC conical–cylindrical combined shells supported by an elastic foundation were investigated employing the HWDM. By means of the HWDM, the governing equations of each shell segment, as well as the continuity and boundary conditions, were converted into a system of algebraic equations. Then, the natural frequencies and modes of the joined shell were achieved by calculating the resultant algebraic equations. The convergence and comparison studies demonstrated that the proposed HWDM has stable convergence, high efficiency, and excellent accuracy. Furthermore, detailed parametric studies were conducted to evaluate the influences of the distribution type and volume fraction of CNTs, foundation stiffness, boundary conditions, and geometric parameters on the natural frequencies of the CCCS. The mode shapes of the CCCS were also presented.

It was found that the CCCS with the FG-X pattern exhibits the highest natural frequencies, while the FG-O shells exhibit the lowest. The natural frequencies increase with an increase in the volume fraction of CNTs. The natural frequencies are significantly influenced by the boundary conditions. Moreover, the increase in the values of the foundation stiffness coefficient within a certain range results in an increased fundamental frequency of the CCCS, and when the stiffness coefficient values reach a certain value, the effects of the foundation stiffness on the fundamental frequency become insensitive. The fundamental frequency increases with a rise in the thickness-to-radius ratio or a decrease in the length-to-radius ratio. The complex variation relationship between the fundamental frequency and semi-vertex angle was observed.

This investigation provides fundamental insights into the vibrational characteristics of FG-CNTRC CCCSs supported by elastic foundations.

## Figures and Tables

**Table 1:** The material properties of (10, 10) armchair single-walled CNTs.

$E_{11}^{CNT}$ (TPa)	$E_{22}^{CNT}$ (TPa)	$G_{12}^{CNT}$ (TPa)	$\rho^{CNT}$ ($\mathrm{kg}/m^{3}$)	$v_{12}^{CNT}$
5.6466	7.0800	1.9445	1400	0.175

**Table 2:** Non-dimensional frequencies $\Omega_{nm}$ of the C-C FG-X CCCS with different J (number in the parenthesis denotes the estimated converged order).

Mode No.		The Maximal Level of Resolution J					
n	m	J=3	J=4	J=5	J=6	J=7	J=8
1	1	0.6496	0.6363	0.6327	0.6318	0.6316	0.6315
				(1.946)	(1.972)	(1.984)	(2.019)
	2	0.8563	0.8476	0.8454	0.8448	0.8447	0.8446
				(1.989)	(1.997)	(2.006)	(1.998)
	3	1.3396	1.2875	1.2738	1.2703	1.2694	1.2692
				(1.938)	(1.982)	(1.995)	(2.010)
2	1	0.5681	0.5512	0.5467	0.5456	0.5453	0.5452
				(1.936)	(1.977)	(1.989)	(2.022)
	2	0.6770	0.6618	0.6579	0.6570	0.6567	0.6566
				(1.987)	(1.996)	(2.008)	(1.959)
	3	1.1882	1.1255	1.1093	1.1052	1.1042	1.1039
				(1.948)	(1.987)	(1.994)	(2.009)

**Table 3:** First 5 frequencies (Hz) of the isotropic CCCSs resting on elastic foundations with different boundary conditions.

Foundation Stiffnesses	Frequencies (Hz)	C-C		C-F		SD-SD	
		Present	ANSYS	Present	ANSYS	Present	ANSYS
$K_{w}=0,\;K_{s}=0$	$f_{1}$	195.14	195.09	76.08	75.87	192.70	192.91
	$f_{2}$	204.98	204.72	86.81	86.64	201.50	201.37
	$f_{3}$	214.34	214.30	125.44	125.46	212.88	212.87
	$f_{4}$	221.28	221.38	172.97	173.02	216.47	216.59
	$f_{5}$	231.10	231.30	210.31	210.32	226.68	226.95
$K_{w}=2\times 10^{3},\;K_{s}=0$	$f_{1}$	224.00	224.14	129.82	129.95	222.10	222.16
	$f_{2}$	231.92	232.09	130.30	130.42	228.97	229.11
	$f_{3}$	241.17	241.22	166.13	166.11	239.89	239.96
	$f_{4}$	247.57	247.44	205.07	205.03	243.30	243.24
	$f_{5}$	256.59	256.43	237.65	237.64	252.68	252.65
$K_{w}=2\times 10^{3},\;K_{s}=10^{3}$	$f_{1}$	417.08	417.24	271.63	271.25	415.27	415.47
	$f_{2}$	430.41	430.30	279.57	279.44	428.44	428.56
	$f_{3}$	480.03	480.25	393.23	393.45	477.61	477.68
	$f_{4}$	493.78	493.56	477.22	477.13	492.94	492.96
	$f_{5}$	526.45	526.10	508.98	508.44	523.14	523.34

**Table 4:** Comparison of the non-dimensional frequency parameters $\Omega_{nm}$ for the F-C homogeneous isotropic CCCS ($K_{w}=K_{s}=0$).

Mode No.		ANSYS FEM	Ma et al. [51]	Efraim and Eisenberger [52]	Caresta and Kessissoglou [44]	Present
n	m					
0	1	0.5026	0.5038	0.5038	0.5054	0.5038
	2	0.6099	0.6098	0.6099	0.6098	0.6098
1	1	0.2928	0.2929	0.2929	0.2934	0.2929
	2	0.6344	0.6358	0.6358	0.6368	0.6358
2	1	0.0998	0.0999	0.1000	0.1000	0.1000
	2	0.5019	0.5026	0.5027	0.5028	0.5027
3	1	0.0874	0.0876	0.0876	0.0873	0.0876
	2	0.3903	0.3915	0.3916	0.3915	0.3916
4	1	0.1445	0.1446	0.1446	0.1445	0.1446
	2	0.3299	0.3303	0.3304	0.3302	0.3304

**Table 5:** First four non-dimensional natural frequencies $\Omega_{i}(i=1\sim 4)$ of FG-CNTRC CCCSs with and without an elastic foundation.

$V_{CNT}^{*}$	Types	($K_{w}=0,\;K_{s}=0$)				($K_{w}=10^{4},\;K_{s}=5\times 10^{3}$)			
		$\Omega_{1}$	$\Omega_{2}$	$\Omega_{3}$	$\Omega_{4}$	$\Omega_{1}$	$\Omega_{2}$	$\Omega_{3}$	$\Omega_{4}$
0.12	UD	0.3332	0.3352	0.3437	0.3467	0.5672	0.6804	0.8360	0.9812
	FG-X	0.3585	0.3597	0.3668	0.3723	0.5702	0.6831	0.8410	0.9892
	FG-O	0.3049	0.3109	0.3135	0.3270	0.5670	0.6805	0.8322	0.9733
	FG-V	0.3266	0.3299	0.3370	0.3443	0.5673	0.6817	0.8354	0.9779
	FG-A	0.3090	0.3132	0.3194	0.3277	0.5682	0.6806	0.8331	0.9754
0.17	UD	0.4207	0.4244	0.4340	0.4407	0.7167	0.8317	0.9540	1.0592
	FG-X	0.4546	0.4554	0.4670	0.4720	0.7238	0.8391	0.9661	1.0733
	FG-O	0.3870	0.3961	0.3970	0.4179	0.7181	0.8327	0.9457	1.0476
	FG-V	0.4145	0.4198	0.4278	0.4399	0.7189	0.8348	0.9517	1.0544
	FG-A	0.3935	0.4000	0.4069	0.4203	0.7202	0.8337	0.9490	1.0516
0.28	UD	0.4676	0.4684	0.4817	0.4823	0.7510	0.8661	0.9849	1.0828
	FG-X	0.5185	0.5239	0.5273	0.5449	0.7726	0.8892	1.0110	1.1093
	FG-O	0.4222	0.4303	0.4319	0.4519	0.7659	0.8802	0.9825	1.0726
	FG-V	0.4568	0.4608	0.4710	0.4792	0.7671	0.8827	0.9898	1.0808
	FG-A	0.4413	0.4462	0.4555	0.4646	0.7682	0.8821	0.9883	1.0795

**Table 6:** Effects of the boundary conditions on the non-dimensional fundamental frequencies $\Omega_{1}$ of CNTRC CCCSs with FG-X pattern resting on elastic foundations.

9	$K_{s}$	Boundary Conditions							
		C-C	SD-SD	SD-C	C-SD	C-F	F-C	SD-F	F-SD
0	0	0.5336	0.4546	0.4988	0.4559	0.0462	0.1666	0.0327	0.0105
	5 × 10^2^	0.6848	0.6142	0.6570	0.6152	0.1150	0.2892	0.0700	0.0520
	1 × 10^3^	0.7709	0.6989	0.7452	0.7071	0.1289	0.3701	0.0915	0.0724
5 × 10^3^	0	0.5693	0.4960	0.5367	0.4973	0.1666	0.2411	0.1359	0.0683
	5 × 10^2^	0.7126	0.6451	0.6854	0.6460	0.1764	0.3373	0.1482	0.0848
	1 × 10^3^	0.7955	0.7037	0.7703	0.7337	0.1857	0.4086	0.1593	0.0983
1 × 10^4^	0	0.6029	0.5341	0.5722	0.5355	0.2132	0.2973	0.1878	0.0947
	5 × 10^2^	0.7392	0.6745	0.7126	0.6754	0.2209	0.3793	0.1967	0.1070
	1 × 10^3^	0.8193	0.7079	0.7946	0.7594	0.2283	0.4436	0.2052	0.1177

**Table 7:** Fundamental frequency $\Omega_{1}$ of FG-X CNTRC CCCSs resting on elastic foundation for different values of $L_{l}/R_{2}$ and $h/R_{2}$ under various boundary conditions (numbers in parentheses indicate foundation stiffness coefficients $K_{w}$ and $K_{s}$).

$L_{l}/R_{2}$	$h/R_{2}$	C-C		SD-SD		F-C	
		(0, 0)	($5\times 10^{3}$$,\;5\times 10^{2}$)	(0, 0)	($5\times 10^{3}$$,\;5\times 10^{2}$)	(0, 0)	($5\times 10^{3}$$,\;5\times 10^{2}$)
0.5	0.005	0.3851	0.4857	0.3578	0.4613	0.1223	0.2491
	0.01	0.5861	0.7525	0.5059	0.6810	0.2127	0.3693
	0.02	0.9119	1.1726	0.7202	0.7671	0.2673	0.6456
	0.05	1.4102	1.7096	0.7441	0.8822	0.4721	1.0521
1	0.005	0.3596	0.4606	0.3176	0.4264	0.1152	0.2104
	0.01	0.5336	0.7126	0.4547	0.6451	0.1666	0.3374
	0.02	0.8221	1.1045	0.6793	0.7301	0.2165	0.5250
	0.05	1.2517	1.4184	0.7189	0.7974	0.3936	0.8299
2	0.005	0.1830	0.2862	0.1726	0.2791	0.1085	0.1800
	0.01	0.2503	0.4327	0.2304	0.4204	0.1297	0.3078
	0.02	0.3543	0.6672	0.3118	0.5956	0.1879	0.3908
	0.05	0.5774	1.0119	0.4803	0.7357	0.2865	0.7114
5	0.005	0.0861	0.1805	0.0806	0.1780	0.0665	0.1456
	0.01	0.1111	0.2781	0.1053	0.2759	0.0928	0.1903
	0.02	0.1443	0.4025	0.1371	0.3886	0.1278	0.3072
	0.05	0.2059	0.7529	0.1920	0.6935	0.1295	0.6622

## Data Availability

Data will be made available upon request.

## Appendix A

Detailed expressions of the constant coefficients $G_{ijk}$ and ${\tilde{G}}_{ijk}$ for FG-CNTRC conical shell segment are listed in this appendix section.

The coefficients $G_{ijk}$ in Equation (13) are given by:

(A1) $$\begin{array}{l} G_{111}=-\frac{A_{66}n^{2}+A_{22}\sin^{2}\alpha }{R^{2}},\;G_{112}=\frac{A_{11}}{R}\sin\alpha ,\;G_{113}=A_{11},\;G_{121}=\frac{-A_{66}-A_{22}}{R^{2}}n\sin\alpha ,\;\\ G_{122}=\frac{A_{12}+A_{66}}{R}n,\;G_{131}=-\frac{A_{22}}{R^{2}}\sin\alpha \cos\alpha ,\;G_{132}=\frac{A_{12}}{R}\cos\alpha ,\;G_{141}=-\frac{B_{66}n^{2}+B_{22}\sin^{2}\alpha }{R^{2}},\;\\ G_{142}=\frac{B_{11}}{R}\sin\alpha ,\;G_{143}=B_{11},\;G_{151}=\frac{-B_{66}-B_{22}}{R^{2}}n\sin\alpha ,\;G_{152}=\frac{B_{12}+B_{66}}{R}n,\;\\ G_{211}=\frac{-A_{66}-A_{22}}{R^{2}}n\sin\alpha ,\;G_{212}=-\frac{A_{12}+A_{66}}{R}n,\;G_{221}=-\frac{A_{66}\sin^{2}\alpha +A_{22}n^{2}+k_{s}A_{44}\cos^{2}\alpha }{R^{2}},\\ G_{222}=\frac{A_{66}}{R}\sin\alpha ,\;G_{223}=A_{66},\;G_{231}=\frac{-A_{22}-k_{s}A_{44}}{R^{2}}n\cos\alpha G_{241}=\frac{-B_{66}-B_{22}}{R^{2}}n\sin\alpha ,\\ G_{242}=-\frac{B_{12}+B_{66}}{R}n,\;G_{251}=-\frac{B_{66}\sin^{2}\alpha +B_{22}n^{2}}{R^{2}}+\frac{k_{s}A_{44}\cos\alpha }{R},\;G_{252}=\frac{B_{66}}{R}\sin\alpha ,\;G_{253}=B_{66},\\ G_{311}=-\frac{A_{22}\sin\alpha \cos\alpha }{R^{2}},\;G_{312}=-\frac{A_{12}\cos\alpha }{R},\;G_{321}=\frac{-A_{22}-k_{s}A_{44}}{R^{2}}n\cos\alpha ,\\ G_{331}=-\frac{k_{s}A_{44}n^{2}+A_{22}\cos^{2}\alpha }{R^{2}},\;G_{332}=\frac{k_{s}A_{55}\sin\alpha }{R},\;G_{333}=k_{s}A_{55},\;G_{342}=\frac{-B_{12}\cos\alpha }{R}+k_{s}A_{55}\\ G_{341}=\frac{k_{s}A_{55}R\sin\alpha -B_{22}\sin\alpha \cos\alpha }{R^{2}},\;G_{351}=\frac{k_{s}A_{44}nR-B_{22}n\sin\alpha }{R^{2}},\\ G_{411}=-\frac{B_{66}n^{2}+B_{22}\sin^{2}\alpha }{R^{2}},\;G_{412}=\frac{B_{11}}{R}\sin\alpha ,\;G_{413}=B_{11},\;G_{421}=\frac{-B_{66}-B_{22}}{R^{2}}n\sin\alpha ,\\ G_{422}=\frac{B_{12}+B_{66}}{R}n,\;G_{431}=-\frac{B_{22}}{R^{2}}\sin\alpha \cos\alpha ,\;G_{432}=\frac{B_{12}}{R}\cos\alpha -k_{s}A_{55},\\ G_{441}=-\frac{D_{66}n^{2}+D_{22}\sin^{2}\alpha }{R^{2}}-k_{s}A_{55},\;G_{442}=\frac{D_{11}}{R}\sin\alpha ,\;G_{443}=D_{11},\\ G_{451}=\frac{-D_{66}-D_{22}}{R^{2}}n\sin\alpha ,\;G_{452}=\frac{D_{12}+D_{66}}{R}n,\;G_{511}=\frac{-B_{66}-B_{22}}{R^{2}}n\sin\alpha ,\;G_{512}=-\frac{B_{12}+B_{66}}{R}n,\\ G_{521}=-\frac{B_{66}\sin^{2}\alpha +B_{22}n^{2}+k_{s}A_{44}R\cos\alpha }{R^{2}},\;G_{522}=\frac{B_{66}}{R}\sin\alpha ,\;G_{523}=B_{66},\\ G_{531}=\frac{-B_{22}n\cos\alpha }{R^{2}}+\frac{k_{s}A_{44}n}{R}G_{541}=\frac{-D_{66}-D_{22}}{R^{2}}n\sin\alpha ,\;G_{542}=-\frac{D_{12}+D_{66}}{R}n,\\ G_{551}=-\frac{D_{66}\sin^{2}\alpha +D_{22}n^{2}}{R^{2}}-k_{s}A_{44},\;G_{552}=\frac{D_{66}}{R}\sin\alpha ,\;G_{553}=D_{66}, \end{array}$$

The coefficients ${\tilde{G}}_{ijk}$ in Equation (25) can be obtained by the following expressions:

(A2) $${\tilde{G}}_{ij1}=G_{ij1},\;{\tilde{G}}_{ij2}=\frac{G_{ij2}}{L_{c}},\;{\tilde{G}}_{ij3}=\frac{G_{ij3}}{L_{c}^{2}}$$
